# Four‐dimensional dose calculations for dynamic tumour tracking with a gimbal‐mounted linear accelerator

**DOI:** 10.1002/acm2.13265

**Published:** 2021-05-27

**Authors:** Emilie E. Carpentier, Ronan L. McDermott, Emma M. Dunne, Marie‐Laure A. Camborde, Alanah M. Bergman, Tania Karan, Mitchell C. C. Liu, Roy M. K. Ma, Ante Mestrovic

**Affiliations:** ^1^ Department of Physics and Astronomy University of British Columbia Vancouver BC Canada; ^2^ Department of Medical Physics BC Cancer – Vancouver Vancouver BC Canada; ^3^ Radiation Oncology BC Cancer Vancouver Vancouver BC Canada

**Keywords:** 4D dose calculation, dynamic tumor tracking, gimballed linac system

## Abstract

**Purpose:**

In this study we present a novel method for re‐calculating a treatment plan on different respiratory phases by accurately modeling the panning and tilting beam motion during DTT (the “rotation method”). This method is used to re‐calculate the dose distribution of a plan on multiple breathing phases to accurately assess the dosimetry.

**Methods:**

sIMRT plans were optimized on a breath hold computed tomography (CT) image taken at exhale (BH_exhale_) for 10 previous liver stereotactic ablative radiotherapy patients. Our method was used to re‐calculate the plan on the inhale (0%) and exhale (50%) phases of the four‐dimensional CT (4DCT) image set. The dose distributions were deformed to the BH_exhale_ CT and summed together with proper weighting calculated from the patient’s breathing trace. Subsequently, the plan was re‐calculated on all ten phases using our method and the dose distributions were deformed to the BH_exhale_ CT and accumulated together. The maximum dose for certain organs at risk (OARs) was compared between calculating on two phases and all ten phases.

**Results:**

In total, 26 OARs were examined from 10 patients. When the dose was calculated on the inhale and exhale phases six OARs exceeded their dose limit, and when all 10 phases were used five OARs exceeded their limit.

**Conclusion:**

Dynamic tumor tracking plans optimized for a single respiratory phase leave an OAR vulnerable to exceeding its dose constraint during other respiratory phases. The rotation method accurately models the beam’s geometry. Using deformable image registration to accumulate dose from all 10 breathing phases provides the most accurate results, however it is a time consuming procedure. Accumulating the dose from two extreme breathing phases (exhale and inhale) and weighting them properly provides accurate results while requiring less time. This approach should be used to confirm the safety of a DTT treatment plan prior to delivery.

## 1 INTRODUCTION

The effective management of intrafractional respiratory motion during radiation therapy treatments is critical to achieving sufficient target coverage while sparing healthy tissue nearby.^1^ Precision of treatment is even more crucial when treating with stereotactic ablative radiotherapy (SABR), a technique which delivers a high conformal biologically effective dose (BED) to the target while the steep dose gradient allows dose to be differentially steered away from adjacent organs at risk (OAR). Given the steep dose gradient of SABR, the dosimetric consequences of physiological organ and target movement can impact the actual received dose to the planning target volume (PTV) and OAR with potential clinical consequences. This is particularly important when treating abdominal lesions with SABR due to the degree of motion in this region.^2^ For example, liver tumors can move up to several centimeters during respiration.^3^ Real‐time dynamic tumor tracking (DTT) is a motion management technique in which the radiation beam follows the target and continuously irradiates it. Tumor tracking offers advantages over other motion management techniques, such as a higher duty cycle compared to gating^4^ and smaller treatment margins compared to a motion encompassing technique.^5^ There are several different options for tumor tracking including moving the multi‐leaf collimators (MLCs)^6^ or couch,^7^ or by using a robotic^8^ or gimballed^9^ linac.

**Fig. 1:**
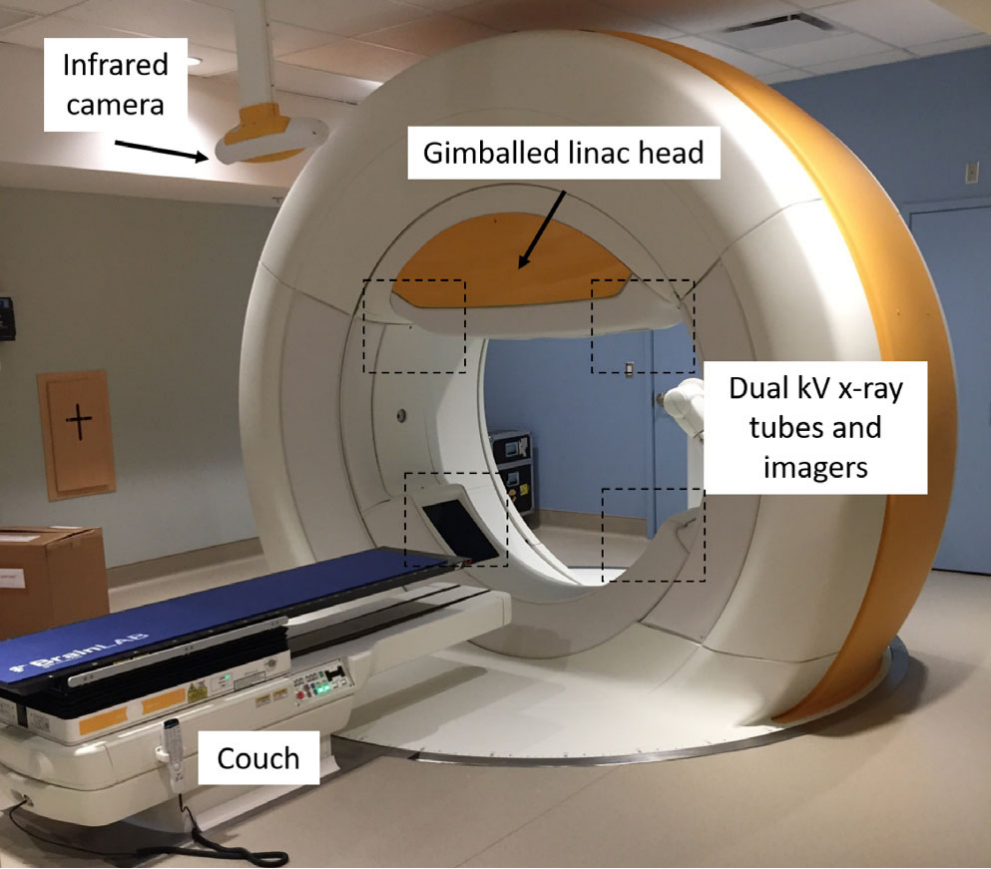
The Vero linear accelerator at BC Cancer – Vancouver. The linac head is mounted on a gimbal system for panning and tilting the beam. The Vero can rotate the gantry around the patient’s superior‐inferior axis and the ring around the anterior‐posterior axis for non‐coplanar beam deliveries. The machine is equipped with two orthogonal kV imagers at ±45° angles to the clinical beam, an infrared camera for capturing the patient’s abdominal motion during respiration, and a 5 degree‐of‐freedom couch (with a sixth degree‐of‐freedom from the ring rotation).

The Vero4DRT (Vero) linear accelerator (linac), shown in Fig. 1, is a gimballed linac capable of DTT by panning and tilting the beam up to ±2.4° in either direction to follow a moving tumor in cranio‐caudal and lateral directions, reaching any point within ±4.2cm in the plane at isocenter perpendicular to the beam.^10^ DTT is dependent on building a four‐dimensional (4D) respiration correlation model prior to each fraction using synchronously monitored internal fiducial markers located near the target and external infrared (IR) markers placed on the abdomen at the point where respiratory motion is the largest.^9^, ^11^, ^12^ Throughout treatment, the motion of the external IR markers is monitored and the 4D respiration correlation model uses that information to determine the real‐time motion of the internal fiducial markers. The system pans/tilts the beam according to the internal marker motion assuming the target’s displacement is identical. The tracking error between the gimbal’s position and the detected marker positions by the orthogonal kV imagers was found to be below 3.08 mm on average and the tracking system has similar performance to other clinical systems that use real‐time tumor tracking.^13^ The linac head is installed on an O‐ring shaped gantry that can rotate around the patient’s inferior‐superior axis and posterior‐anterior axis, enabling non‐coplanar beam deliveries specified by a gantry and ring angle, respectively. The Vero also has two integrated orthogonal sets of kV x‐ray tubes and flat panel detectors for on‐board imaging.^9^, ^11^, ^12^

The RayStation (RaySearch Laboratories, Sweden) treatment planning system (TPS) is commercially available for generating plans for delivery on the Vero.^14^, ^15^ However, it does not incorporate the panning and tilting beam geometry that occurs during DTT when calculating a dose distribution for a plan. Treatment plans are created, optimized, and evaluated on a single respiratory phase computed tomography (CT) image only. This neglects two differences among respiratory phases: (a) The source‐to‐target and source‐to‐surface distances can change as the beam pans and tilts, and (b) the variable distance between OARs and the target during respiration. Therefore, the expected dose to an OAR as calculated by the TPS may be inaccurate. Three‐dimensional conformal radiation therapy (3DCRT), step‐and‐shoot intensity modulated radiation therapy (sIMRT), and dynamic conformal arcs are the only treatment techniques on the Vero that can have DTT enabled. Other treatment techniques, such as dynamic IMRT and volumetric modulated arc therapy have considerable leaf motion while the beam is on which can accentuate inaccuracies in the dose distribution. Therefore DTT with these treatment techniques is not available.

Other groups have addressed the limitations of planning tracked treatments on one phase by re‐calculating the dose distribution on other respiratory phases. Since the TPS cannot pan/tilt the beam on these other phases, they have either simplified the beam motion as a translation within the TPS^16^ (referred to as the *translation method*), or properly modeled panning/tilting by performing the dose calculation external to the TPS.^17^, ^18^ We have developed a novel approach for modeling the panning and tilting beam motion during DTT in the TPS to calculate the dose distribution of a plan on other respiratory phases. By individually altering the gantry, ring, and collimator angles, as well as shifting the patient, for each beam in a 3DCRT or sIMRT plan we can re‐create the path of the beam when it pans/tilts during that respiratory phase. Compiling these new beams into a plan on a different respiratory phase CT allows us to re‐calculate the dose distribution while accurately modeling the rotated beam during DTT. We refer to this method of re‐calculating the dose distribution on another respiratory phase as the *rotation method*.

In this paper, we describe the rotation method for re‐calculating a dose distribution on any respiratory phase. The advantage of our rotation method is that the user stays in the TPS to perform the calculation. The procedure is more efficient and streamlined and uses clinically commissioned beam data to perform the dose calculation. It also minimizes errors that may arise from transferring data to another application. To our knowledge, this is the first time such an approach has been mentioned in the literature for a gimballed linac. This rotation method is demonstrated here for dose calculations with the Vero, but the ideas and concepts can be applied to any other system that pans/tilts the beam during tracking.

## 2 MATERIALS AND METHODS

A retrospective study was conducted to compare the dose to OARs when the dose distribution is calculated on different respiratory phases using two different methods (translational and rotational) and to demonstrate how the calculation method can affect clinical decisions. This study also investigated the temporal resolution required to retrieve accurate dosimetric information.

### 2.1 4DCT phase data

Patients treated with liver SABR at our center have three or more gold fiducials implanted near their tumor prior to a planning CT simulation scan. A patient’s respiratory trace is recorded using Varian’s *RPM* system during their 4DCT scan and thereafter retrospectively binned into ten respiratory phases in external software (Advantage, GE). Clinical plans are generated on breath‐hold images taken at exhale (BH_exhale_). This study uses a patient’s 4DCT data to evaluate doses from a plan optimized on the BH_exhale_ CT on multiple breathing phases.

### 2.2 Translation method

The translation method used in this study is described by Depuydt et al.^16^ CT images representing two different respiratory phases are rigidly registered by aligning the fiducial markers implanted near the target in each image. A plan optimized on the BH_exhale_ CT is transferred to the inhale (0%) phase of the 4DCT based on this registration and the dose distribution is re‐calculated. This models tumor tracking as a translation of the beam, and all beams are shifted by the same amount.

### 2.3 Rotation method

**Fig. 2:**
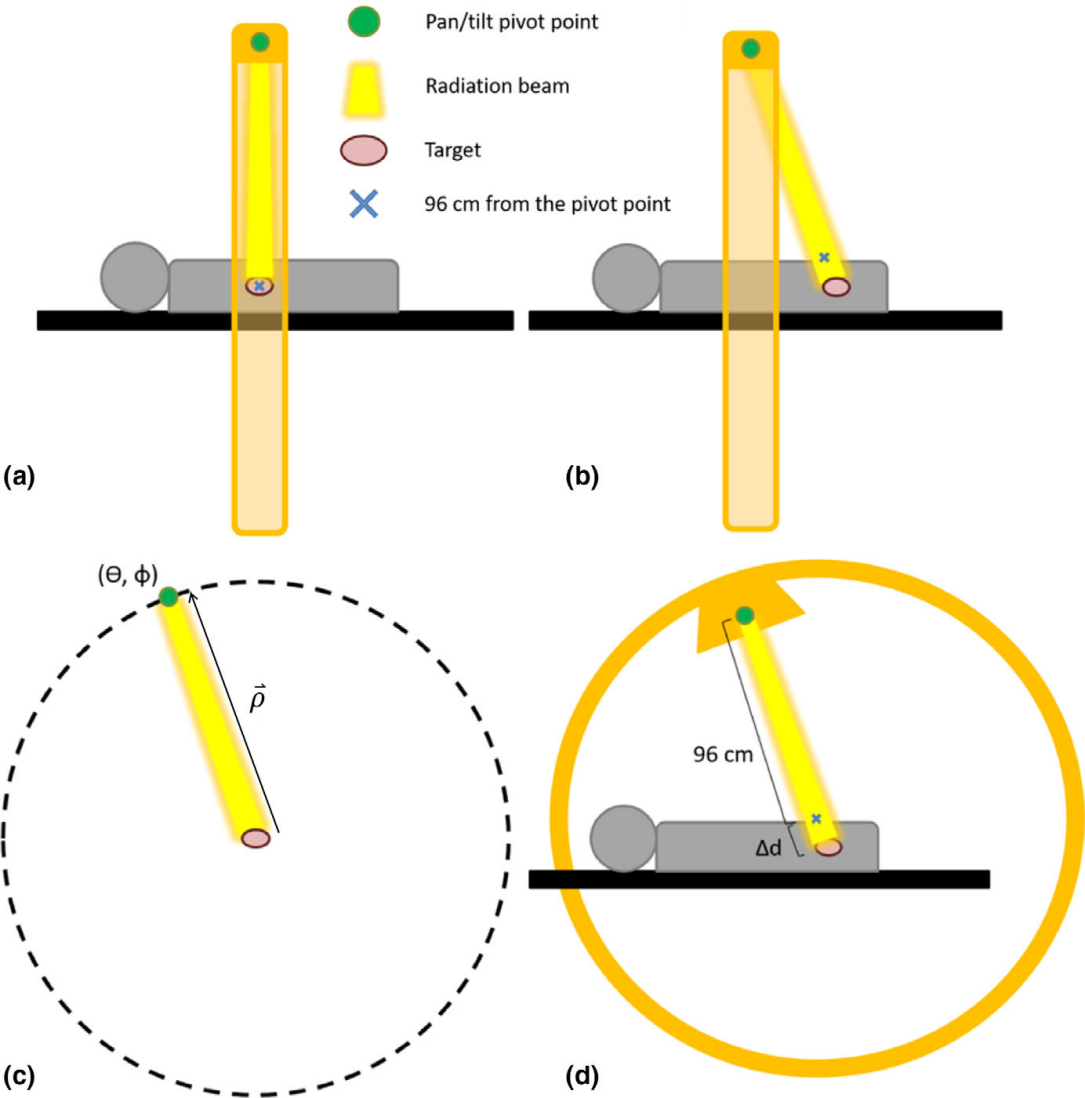
A schematic demonstrating an example of how the rotation method re‐calculates the gantry and ring angles, as well as a patient shift for each beam based on the tumor’s motion during respiration. (a) A side view of the patient on the couch with the Vero at gantry and ring angles of 0 degrees. The machine is translucent for visualizing the setup. The patient is currently in their exhale respiratory phase. (b) When the patient inhales during treatment, the target moves inferiorly and the beam rotates to follow it. Now, the beam path to the target is greater than 96 cm. (c) The new relative location of the target to the pivot point in spherical coordinates (θ,φ,ρ). (d) Spherical coordinates are translated into new gantry and ring angles, and a patient shift. The collimator angle will also need to be adjusted in the TPS to maintain the appropriate BEV (not illustrated here).

This novel method re‐creates the beam’s path through the patient when it pans and tilts by changing the gantry, ring, and collimator angles of the Vero, as well as the location of each beam's isocenter, within the TPS. Figure 2 demonstrates this concept. The pivot point for panning and tilting the beam is 96 cm from the isocenter, and the isocenter is at the center of the Vero ring. Therefore, the gantry and ring angles define polar and azimuthal coordinates on a sphere of radius 96 cm with isocenter coinciding with the sphere’s center and the pivot point along the sphere’s surface. The patient shift is necessary to account for the different beam path length to the target when it pans/tilts. The collimator angle on the Vero cannot rotate for treatment by design, but with this method it must be rotated in the TPS to maintain the correct beam’s eye view (BEV) after changing the gantry and ring angles.

A script was written within the RayStation TPS to perform this calculation for each beam in the original plan and copy the new beams to a plan on a different respiratory phase. The dose distribution is then re‐calculated. This allows the dosimetry for a plan to be verified on a different respiratory phase than what it was originally optimized for while modeling the appropriate beam geometry. This process was tested on a cylindrical phantom with a cylindrically shaped target that was moved 1–2 cm in one, two, and three dimensions in the TPS to simulate different complexities of respiratory motion.

### 2.4 Dose re‐calculation

A retrospective analysis was performed with the CT data of 10 patients previously treated with liver SABR with fiducial markers implanted in the liver adjacent to the target. OARs of interest previously delineated on the BH_exhale_ CT were rigidly propagated to all other phases of their 4DCT and reviewed and adjusted as necessary by oncologists. A sIMRT plan was optimized on the BH_exhale_ CT in the RayStation TPS following clinical protocols, meeting the prescription and dose limit requirements for each patient.

#### 2.4.1 Dose re‐calculation on the inhale (0%) phase

**Fig. 3:**
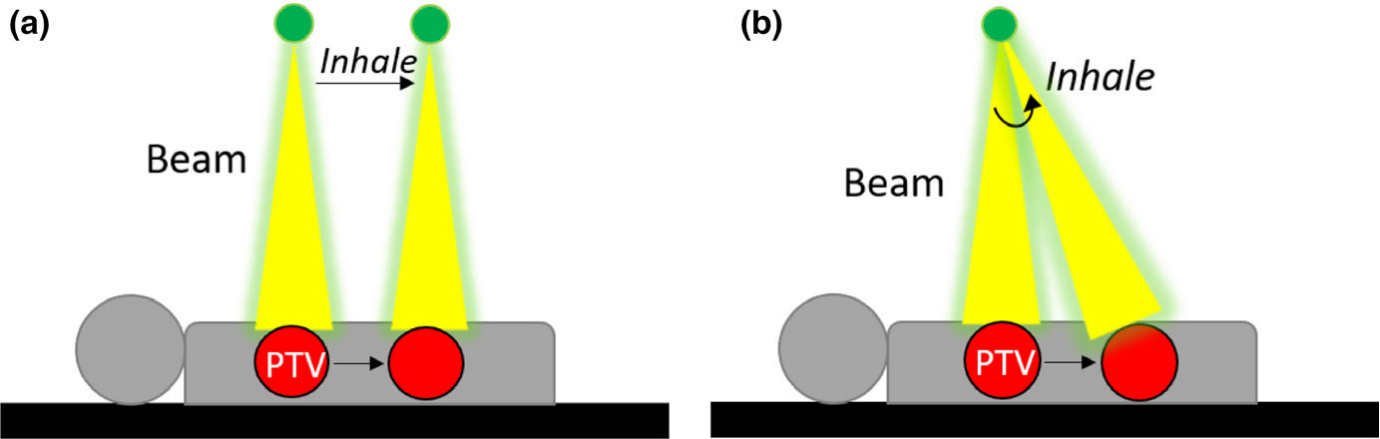
This schematic shows a sagittal view of a patient on the treatment couch. The PTV moves inferiorly when the patient inhales. (a) The simplified translation method models DTT as a translation of the beam to follow the moving tumor. (b) Our rotation method correctly models the panning/tilting beam motion during DTT where the beam pivots to follow the moving tumor.

These plans were transferred to the inhale (0%) phase CT using both the translation and rotation methods outlined above. The maximum dose to OARs of interest on the inhale phase, as calculated by each method, was reported. The schematic in Fig. 3 demonstrates the difference between the translation and rotation methods in the TPS.

#### 2.4.2 Dose re‐calculation on multiple phases

During a DTT treatment, the plan is not delivered statically in the exhale or inhale position, so the next step was to model the plan being delivered throughout the breathing cycle and incorporate a temporal element. Dose distributions calculated on other breathing phases were transferred back to the BH_exhale_ CT using the ANACONDA deformable image registration (DIR) algorithm^19^ available within RayStation. The OARs of interest for each patient were used as controlling regions of interest (ROIs) to improve the optimization.

**Fig. 4:**
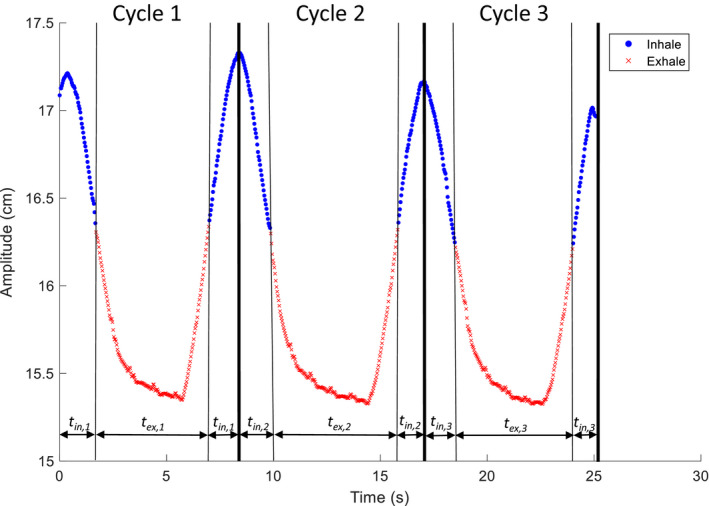
The breathing trace for a patient acquired during their 4DCT scan. To determine the weighting for the inhale and exhale phases, the breathing trace is divided into cycles between points of maximum inhalation. The halfway point between maximum and minimum amplitude for each cycle is calculated. The time spent above the halfway amplitude (*t_in_*) is considered “inhale” and the time below this point (*t_ex_*) is considered “exhale”. The average inhale and exhale weighting is calculated from all the complete cycles available in the breathing trace.

First, the plan was re‐calculated on the inhale (0%) and exhale (50%) breathing phases from the 4DCT using only the rotation method. These dose distributions were then deformed back to the BH_exhale_ CT image. In this study the BH_exhale_ CT image was always the reference image for dose accumulation. The patient’s breathing trace, taken at the time of their 4DCT, was divided into the “inhale” and “exhale” halves of their breathing cycle based on their breathing amplitude. The percentage of their total breathing time spent in either phase was calculated, and the “inhale” and “exhale” phase weightings were used to weight the dose distributions from the inhale (0%) and exhale (50%) CT images after they were deformed to the BH_exhale_ CT and summed together. Figure 4 shows how the breathing trace is divided to determine the weighting for each phase.

Second, the plan was re‐calculated on all 10 breathing phases of the 4DCT and these were deformed to the BH_exhale_ CT and summed together with equal weightings since each image phase represents the same duration of time. As such, the proper phase weighting is inherently included in this approach.

To summarize, we have four different scenarios that re‐calculate the dose to organs at risk during DTT. Table 1 summarizes the four methods for dose re‐calculation outlined above and will be referenced throughout the rest of this work. With increased accuracy in each dose re‐calculation method also comes increased complexity and time to perform the calculation. The maximum dose to OARs of interest was reported under these scenarios to compare what degree of complexity is needed to maintain accuracy in dose calculations for DTT during sIMRT plans.

**Table 1:** A summary of the dose calculation methods used in this study. Original_BH_ is the original dose distribution calculated following our current planning procedures.

Method	Description
Original_BH_	The original plan optimized on the BH_exhale_ CT (a breath‐hold image taken at exhale).
Inhale_trans_	The original plan is re‐calculated on the inhale (0%) phase of the 4DCT using the **translation** method.
Inhale_rot_	The original plan is re‐calculated on the inhale (0%) phase of the 4DCT using the **rotation** method.
Sum_2Phases_	The original plan is re‐calculated on the inhale (0%) and exhale (50%) phases of the 4DCT using the **rotation** method. These dose distributions are deformed to the BH_exhale_ CT and summed together. The weighting for the 2 phases is calculated from the patient’s breathing trace.
Sum_10Phases_	The original plan is re‐calculated on all 10 breathing phases from the 4DCT using the **rotation** method. All 10 dose distributions are deformed to the BH_exhale_ CT and summed together (which inherently models proper phase weighting).

## 3 RESULTS

### 3.1 Rotation method

The script to re‐calculate a plan on another respiratory phase using the rotation method can be executed in 2‐3 minutes for a 7‐field sIMRT plan. The formulae used to re‐calculate the new gantry ($G_{2}$), ring ($R_{2}$), and collimator ($C_{2}$) angles, and patient shift (x,y,z), are given by eqs. ((1), (2), (3), (4a), (4b), (4c), (5a), (5b), (5c), (6)) respectively.

(1) $$G_{2}=\tan^{‐1}\left(\frac{\sqrt{{\left[96\sin\;G_{1}\;\cos\left(‐R_{1}\right)‐\Delta fid_{x}\right]}^{2}+{\left[96\sin G_{1}\;\sin\;\;\left(‐R_{1}\right)‐\Delta fid_{y}\right]}^{2}}}{96\cos G_{1}‐\Delta fid_{z}}\right)$$

(2) $$R_{2}=‐\tan^{‐1}\left(\frac{96\;\;\sin G_{1}\;\;\sin\left(‐R_{1}\right)‐\Delta fid_{y}}{96\;\;\sin G_{1}\;\;\cos\left(‐R_{1}\right)‐\Delta fid_{x}}\right)$$

(3) $$C_{2}=\left\{\begin{array}{cc} R_{2}‐R_{1}, & G_{2}>270\;\text{or}\;G_{2}<90\\ ‐\left(R_{2}‐R_{1}\right), & 90<G_{2}<270\\ 0, & G_{1}=90\\ 180, & G_{1}=270 \end{array}\right.$$

(4a) $$x=\left(96\;\;\sin G_{1}\;\cos\left(‐R_{1}\right)+fid_{1x}\right)‐\frac{96\;\rho_{x}}{\sqrt{\rho_{x}^{2}+\rho_{y}^{2}+\rho_{z}^{2}}}$$

(4b) $$y=\left(96\;\;\sin G_{1}\sin\left(‐R_{1}\right)+fid_{1y}\right)‐\frac{96\;\rho_{y}}{\sqrt{\rho_{x}^{2}+\rho_{y}^{2}+\rho_{z}^{2}}}$$

(4c) $$z=\left(96\;\cos G_{1}\;+\;fid_{1z}\right)‐\frac{96\;\rho_{z}}{\sqrt{\rho_{x}^{2}+\rho_{y}^{2}+\rho_{z}^{2}}}$$

The vector $\vec{\rho }=\left(\rho_{x},\rho_{y},\rho_{z}\right)$ in Fig. 2(c) shown by [eqs. 5(a)–5(c)] describes the path of the beam from the pivot point to the target and $\left(\Delta fid_{x},\Delta fid_{y},\Delta fid_{z}\right)$ given by eq. (6) is the displacement of the fiducial markers between the two respiratory phases:

(5a) $$\rho_{x}=96\;\sin G_{1}\;\cos\left(‐R_{1}\right)+2fid_{2x}‐iso_{x}‐fid_{1x}$$

(5b) $$\rho_{y}=96\;\sin G_{1}\;\sin\left(‐R_{1}\right)+2fid_{2y}‐iso_{y}‐fid_{1y}$$

(5c) $$\rho_{z}=96\;\cos G_{1}\;+\;2fid_{2z}‐iso_{z}‐fid_{1z}$$

(6) $$\left(\Delta fid_{x},\Delta fid_{y},\Delta fid_{z}\right)=\left(fid_{2x}‐fid_{1x},fid_{2y}‐fid_{1y},fid_{2z}‐fid_{1z}\right)$$

These equations are functions of the following variables:

- the position of the fiducial markers on the original respiratory phase $\left(fid_{1x},fid_{1y},fid_{1z}\right)$ and the new respiratory phase $\left(fid_{2x},fid_{2y},fid_{2z}\right)$
- the location of the center of the PTV on the original image used for planning (the original isocenter): $\left(iso_{x},iso_{y},iso_{z}\right)$
- the original gantry and ring angles for each beam: $G_{1}$ and $R_{1}$.

​

**Fig. 5:**
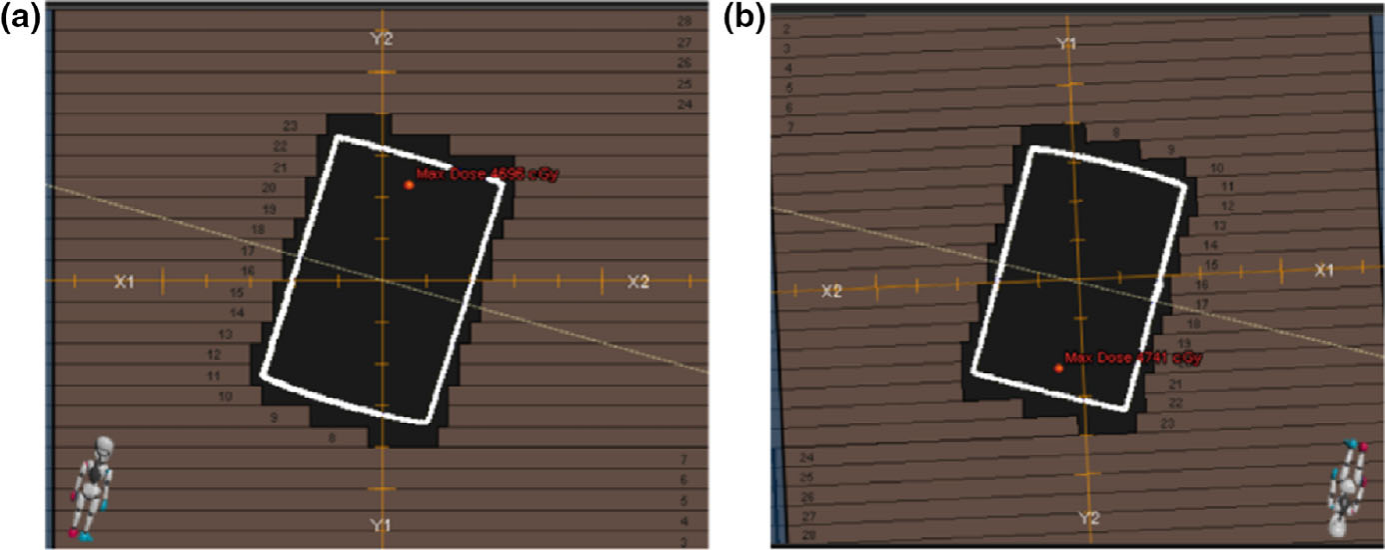
(a) BEV of the cylindrical target during the “exhale” phase. The leaves are conformal to the target shape. In the original plan, the collimator angle is always at 0 degrees. (b) BEV for the same beam after its gantry, ring and collimator angles, as well as isocenter shift, are re‐calculated to simulate tracking on the “inhale” phase when the target moves 0.46cm left, 1.09 cm inferior, and 0.99 cm posterior. The collimator is now rotated 182.5 degrees because of the changed gantry and ring angles. The leaves form the same shape around the target as they did on the “exhale” phase.

Testing the script on a sIMRT plan optimized for a cylindrical phantom produced the new beam parameters shown in Table 2 when the cylindrical‐shaped target moved 0.46 cm left, 1.09 cm inferior, and 0.99 cm posterior to simulate respiratory motion. The beam’s eye view (BEV) for each beam in the newly calculated plan was compared with the BEV for the same beam in the original plan to ensure the target is maintained in the center of the aperture. As an example, this is shown for beam 1 in Fig. 5.

**Table 2:** An example of the beam parameters for a sIMRT plan optimized for a cylindrical phantom whose target was moved 0.46 cm left, 1.09 cm inferiorly, and 0.99 cm posteriorly to simulate respiratory motion. For each beam in the plan, the original (exhale phase) isocenter location, gantry angle, ring angle, and collimator angle are shown followed by the newly calculated (inhale phase) isocenter location, gantry angle, ring angle and collimator angle. The new isocenter is different for each beam to compensate for the changing ​beam path length during tracking.

Beam #	Original isocenter (x,y,z) (cm)	Original gantry angle (^o^)	Original ring angle (^o^)	Original collimator angle (^o^)	New isocenter (x,y,z) (cm)	New gantry angle (^o^)	New ring angle (^o^)	New collimator angle (^o^)
1	−0.11, −87.84, 28.01	192	17	0	−0.74, −86.79, 28.20	168.5	194.5	182.5
2	−0.11, −87.84, 28.01	234	343	0	−0.94, −86.95, 28.72	126.5	162.2	180.8
3	−0.11, −87.84, 28.01	270	17	0	0.13, −87.05, 29.01	90.6	196.5	180.0
4	−0.11, −87.84, 28.01	306	343	0	−0.05, −86.66, 28.59	54.4	162.2	179.2
5	−0.11, −87.84, 28.01	342	17	0	−0.24, −86.93, 27.88	17.8	195.3	178.3
6	−0.11, −87.84, 28.01	30	17	0	−0.81, −86.76, 28.60	30.7	18.0	1.0
7	−0.11, −87.84, 28.01	150	0	0	−0.04, −86.85, 28.05	150.0	1.2	358.8

### 3.2 Dose re‐calculation on the inhale (0%) phase

**Fig. 6:**
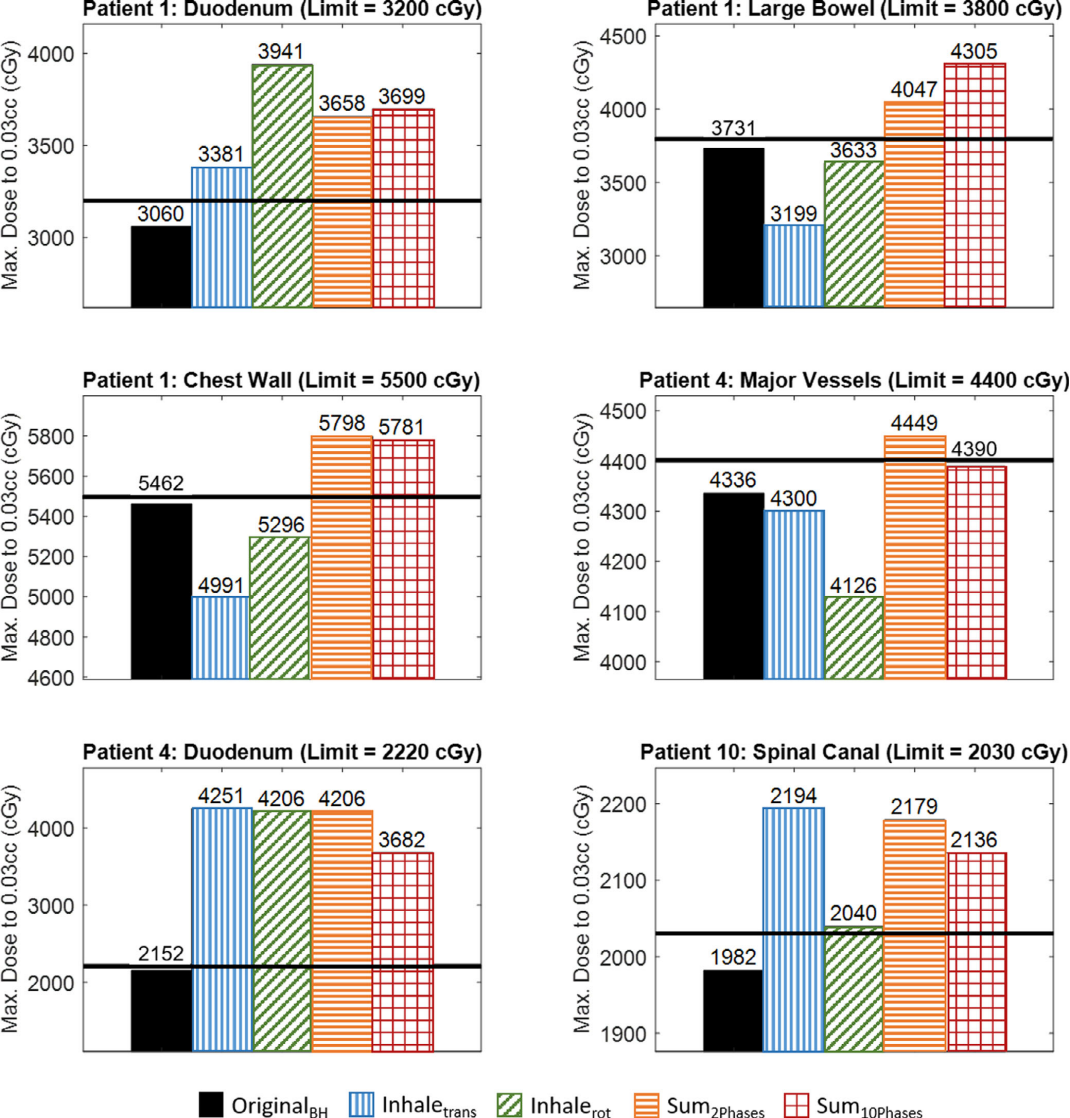
This plot shows the maximum dose to 0.03cc of the six organs that met their dose limit when the plan was optimized on the BH_exhale_ image (original method) but exceeded their dose limit when the plan was re‐calculated using a different method. The maximum dose is reported for each of the calculation methods described in Table 1. The horizontal black lines indicate the dose limit for that OAR. The exhale/inhale phase weightings for these three patients were 72%/28% (patient 1), 66%/34% (patient 4), and 65%/35% (patient 10).

**Fig. 7:**
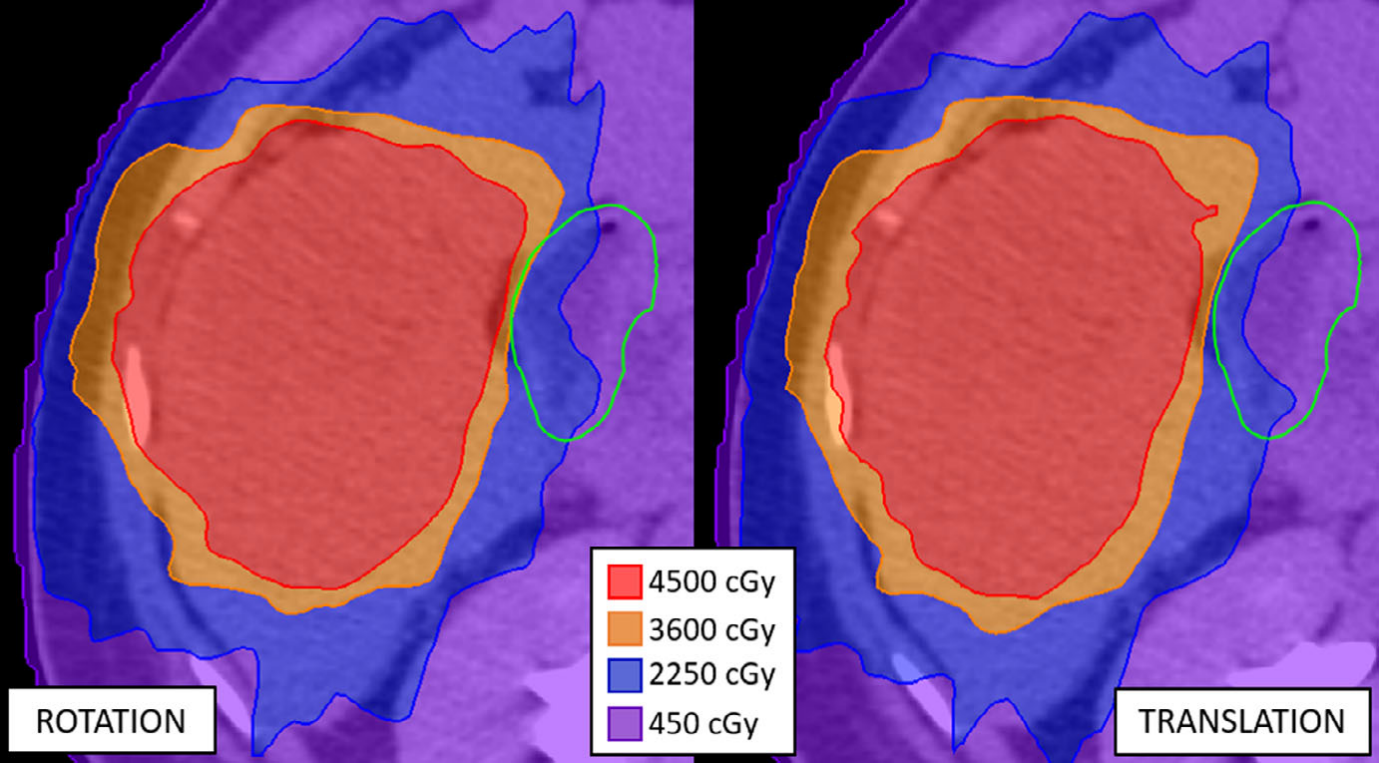
The dose distribution re‐calculated on the inhale phase using the rotation method (left) and the translation method (right) for patient 1. The green contour delineates the duodenum, which has a dose limit of 3200 cGy. The maximum dose to the duodenum was 3941 cGy using the Inhale_rot_ method and 3381 cGy using the Inhale_trans_ method.

When the plan was re‐calculated on the inhale phase using the translation method (Inhale_trans_), 13 out of 26 OARs had a higher maximum dose than from the original plan (Original_BH_). When the plan was re‐calculated on the inhale phase using the rotation method (Inhale_rot_), 14 out of 26 OARs had a higher maximum dose than on the original plan (Original_BH_). In both cases (Inhale_trans_ and Inhale_rot_), three OARs exceeded their dose constraint after meeting their dose limit with Original_BH_. However, the maximum dose calculated between the two methods differed significantly for many of the 26 OARs, as shown in the second and third columns for each OAR plot in Fig. 6. Therefore, when re‐calculating the dose on multiple phases we only used the rotation method as the translation method was found to be inadequate. Figure 7 shows an example of the different dose distributions produced on the inhale CT using the translation and rotation methods.

### 3.3 Dose re‐calculation on multiple phases

Sum_2Phases_ utilizes the patient’s breathing trace data to weight the inhale and exhale dose distributions. The ratio of time spent in each phase ranged from 56%/44% to 74%/26% (exhale/inhale) among the ten patients. When re‐calculating a dose distribution with Sum_2Phases_, six OARs exceeded their dose constraint. Sum_10Phases_ uses the rotation method on all 10 phases of the 4DCT scan. When the dose distribution was re‐calculated using this method, five OARs exceeded their dose constraint. These results are shown in the fourth and fifth columns of each OAR plot in Fig. 6.

## 4 DISCUSSION

Optimizing and calculating a treatment plan on the BH_exhale_ CT only provides dosimetric information representing the entire plan being delivered during that phase of the respiratory cycle. During DTT the plan is delivered throughout the entire respiratory cycle, thus it is important to gather dosimetric information during other phases. Checking the maximum dose to OARs on another respiratory phase can be used to conservatively identify plans that would not be safe for DTT. Clinically at our center, the Inhale_trans_ method is used to check if a plan could be unsafe for DTT prior to treatment.

The translation method is simple to implement but its representation of the beam's path through the body can give inaccurate dosimetric results. The rotation method is more complex but using the scripting environment in the TPS makes the calculation easily accessible. The rotation method accurately models the beam’s path through the body as well as the correct source‐to‐surface and source‐to‐target distances to provide more accurate dosimetry. Using DIR to sum the dose contributions from different respiratory phases gives a more accurate representation of the real dose distribution than assessing the dosimetry on each phase separately (e.g., inhale). If regions of high dose on multiple phases align, the maximum dose to an OAR will remain high. However, if the high dose regions do not overlap, they may sum to a lower dose than either phase alone would predict. This could influence clinical decisions regarding whether a plan is safe for tracking.

Most people do not spend equal time in their exhale and inhale breathing phases. Sum_2Phases_ uses the breathing trace data acquired during the 4DCT to properly determine the weighting for each extreme phase of the breathing cycle (inhale and exhale). Sum_10Phases_ is the most accurate method available to us within the TPS. It also properly models the time spent in each breathing phase by using ten images representing equal segments in time over the entire breathing cycle. However, in order to create accurate image registrations between each phase and the BH_exhale_ image to deform the dose distribution, it requires contouring the OARs of interest on each image. In this study that included 286 contours for all 10 patient’s 26 OARs on 11 images (the 10 phases of the 4DCT and the original BH_exhale_ CT used for planning). This extra time may not be feasible for a clinical workflow. Therefore, based on the results from this study, Sum_2Phases_ is the suggested procedure to follow as it provides results most similar to Sum_10Phases_ (as shown in Fig. 6) while requiring less contouring and calculation time.

Figure 6 demonstrates the risk of making clinical decisions from inaccurate dosimetric results. All six OARs meet their dose constraints from the original plan on the BH_exhale_ phase because the plan was optimized for this phase. At this center, the Inhale_trans_ method is used to check the maximum dose to an OAR of concern before approving a plan for treatment. For three of the six OARs shown in Fig. 6, the Inhale_trans_ method would indicate the OAR is safe for DTT treatment since it is below its dose limit both on the BH_exhale_ image and the inhale (0%) phase, but the Sum_10phases_ method (the most accurate method) shows this OAR would in fact exceed its limit. Even the Inhale_rot_ method, which models the proper beam geometry, would not indicate to a clinician that an OAR will exceed its dose constraint in these cases. This demonstrates the importance of adequately accumulating dose between different breathing phases when making clinical decisions.

Planning on a mid‐ventilation phase of a 4DCT, rather than an extreme end of the respiratory cycle (exhale or inhale), may provide more consistent dosimetric results among all respiratory phases. At this center, planning is done on a breath hold exhale phase CT with contrast to improve tumor visualization. The timing between contrast injection and when the image is taken must be precise. This is more difficult to time with mid‐ventilation during a 4DCT scan than on a breath hold CT, but if contrast is not being used planning on mid‐ventilation is a viable option.

Several other groups have also acknowledged the need for better dosimetry for DTT plans with the Vero and have developed solutions to model the beam motion during treatment. One group uses the translation method by aligning the fiducial markers near the target on each phase of a 4DCT before transferring and re‐calculating the dose distribution on each phase. Then, using deformable image registration they accumulated the dose to one reference respiratory phase for a final total dose distribution.^16^

Other groups properly model the beam motion as a rotation around a pivot point but are required to leave the TPS to perform this dose calculation. Ishihara *et al*. developed a 4D Monte Carlo algorithm to calculate the dose distribution during tumour tracking on the Vero from a ten phase 4DCT.^17^, ^20^ However, they do not deform and accumulate these 10 dose distributions. Prasetio *et al*. model gimbal rotation by rotating each phase’s CT image from a 4DCT outside the TPS based on the gantry, ring, and pan and tilt angles that would occur during that respiratory phase. The rotated CT was then brought back into the TPS for a dose calculation.^18^ While this approach provides an accurate representation of tumor tracking, it requires the user to leave the TPS in order to model the panning and tilting motion. Our rotation method is advantageous because the user stays in the TPS to perform the dose calculation and it is also much more time efficient.

In this study, the Sum_10phases_ method was the most accurate and time consuming. However, it also has a limited temporal resolution that is dependent on the amount of phases in the 4DCT. It has been shown that accumulating dose distributions with a temporal resolution of 500 ms is adequate for an accurate dose reconstruction.^21^ Therefore, if a respiratory cycle has a period of five seconds a ten‐phase 4DCT would provide this resolution.

Using a 4DCT to calculate the dose on other phases is a limitation of our technique because it assumes the patient’s breathing is the same during treatment as it was for their 4DCT. One could use the motion of the fiducial markers during treatment in the calculations above to check if a daily fraction’s dose distribution is consistent with the dose distribution calculated using the 4DCT images. This could identify instances when adaptive offline re‐planning is necessary. Future work will include developing appropriate treatment planning strategies to ensure a plan is safe for tracking and take advantage of respiratory motion to create a more optimal plan. This will incorporate multiple respiratory phases during plan creation and optimization and will require the proper panning/tilting beam geometry to be modeled.

The script that automates the calculation for our rotation method in the TPS makes it easily accessible for clinical use. The correct beam geometry of the rotation method provides more accurate dosimetry than the translation method. While the Sum_10Phases_ method is the most accurate for calculating dose to an OAR, the time it takes to contour on all phases of the 4DCT can make this method clinically infeasible. The Sum_2Phases_ method is a useful compromise as it still produces accurate results and requires less time to implement. Clinical decisions as to whether a treatment plan is safe for DTT are made by checking the dose on other respiratory phases, therefore it is imperative that the dosimetry is as accurate as possible while still being easily accessible.

## 5 CONCLUSION

We have developed a method for re‐calculating the dose distribution of sIMRT plans on different respiratory phases that correctly models the pan/tilt beam geometry during DTT while staying within the TPS. The same method can be used for 3DCRT plans. Correctly modelling DTT beam motion as a rotation when re‐calculating the maximum dose to OARs on a different respiratory phase can provide different outcomes than when the beam motion is simplified to a translation. Accumulating the dose distributions from multiple respiratory phases onto one reference phase and summing them together gives a more accurate calculation of the maximum dose to an OAR. While accumulating the dose from all 10 phases of a 4DCT is the most accurate, it takes a considerable amount of time. Therefore, accumulating the dose from the exhale and inhale phases only, and weighting them according to a patient’s breathing trace, should be used to confirm the safety of a sIMRT plan intended for DTT prior to delivery.

## Author contributions

All authors on this manuscript contributed to the conception of this work or the acquisition, analysis, and interpretation of the data. Everyone was either involved in drafting the work or made critical revisions prior to submission. All authors approved this final version being submitted and agree to be accountable to the work presented.

## Data Availability

The data that support the findings of this study are available on request from the corresponding author. The data are not publicly available due to privacy or ethical restrictions.
